# Advancing targeted axillary dissection in node-positive breast cancer: insights from pooled analyses and systematic review

**DOI:** 10.1093/bjs/znae141

**Published:** 2024-07-02

**Authors:** Umar Wazir, Kefah Mokbel

**Affiliations:** London Breast Institute, The Princess Grace Hospital, London UK; London Breast Institute, The Princess Grace Hospital, London UK

*Dear Editor*

We read with great interest the review article by de Wild *et al*.^1^, wherein the authors systematically reviewed various techniques used in localizing and harvesting pathological lymph nodes as part of targeted axillary dissection (TAD) in patients undergoing neoadjuvant systemic therapy (NST) for node-positive early breast cancer^1^.

We agree with the authors that single-stage localization is more precise than two-stage localization procedures, especially in excellent responders^2^. Furthermore, deploying the active localization marker at the time of biopsy is more cost-effective in the context of public healthcare funding and provision. As the authors reviewed studies incorporating both marked lymph node biopsy (MLNB) and sentinel lymph node biopsy (SLNB), it would have been prudent to report the false-negative rate (FNR) of each component compared with TAD to demonstrate the superiority of both MLNB alone over SLNB alone, given that many centres are using the latter to stage the axilla post-NST in patients with node-positive disease^2^. It is also important to highlight that, despite the systematic nature of their review, the authors overlooked important studies meeting their inclusion criteria, particularly regarding the use of electromagnetic reflectors.

We have recently reported a pooled analysis evaluating radar reflector localization (RRL) in TAD in patients undergoing NST for node-positive early breast cancer^2^.

The pooled analysis of 252 procedures revealed a 99.6% (95% c.i., 98.9 to 100) successful localization rate, 100% retrieval rate and 81% (95% c.i., 76 to 86) concordance rate between SLNB and MLNB. The average duration from RRL deployment to surgery was 52 days (range: 1–202). Pathological complete response (pCR) was observed in 42% (95% c.i., 36 to 48) of cases, with no significant migration or complications reported. Omitting MLNB or SLNB would have understaged the axilla in 9.7% or 3.4% (*P* = 0.03) of patients respectively. This underscores the importance of incorporating MLNB in axillary staging post-NST in initially node-positive patients in line with the updated National Comprehensive Cancer Network (NCCN) guidelines^2^.

In a similar pooled analysis (Alamoodi et al, J. Clin. Med. 2024, 13(10), 2908) of 497 procedures using magnetic seeds (Magseed, Endomagnetics Inc., Cambridge, UK) for localization, we observed a 100% successful deployment rate, 94.2% successful localization rate (95% c.i., 93.7 to 94.7), 98.8% retrieval rate and 68.8% concordance rate (95% c.i., 65.6 to 72.0).

However, we agree that magnetic seeds tend to generate significant MRI artefacts compared to Savi Scout (Merit Medical, Aliso Viejo, CA, USA) reflectors, which could interfere with the radiological assessment of response to NST and require the removal of all metal instruments from the surgical field during localization. Furthermore, we agree with the authors regarding the need for high-quality prospective data to compare the performance of these novel localization systems in TAD.
